# The Value of Early Active Rehabilitation on Long-Term Functional Outcomes in Neer Type-4 Valgus Impacted Proximal Humeral Fractures Treated with Open Reduction and Internal Fixation: A Retrospective Cohort Study

**DOI:** 10.3390/jcm14186660

**Published:** 2025-09-22

**Authors:** Haiyan Zhou, Liming Cheng

**Affiliations:** 1 School of Medicine, Tongji University, Shanghai 200092, China; 2 Department of Orthopaedic Surgery, Yueyang Hospital of Integrated Traditional Chinese and Western Medicine, Shanghai University of Traditional Chinese Medicine, Shanghai 200043, China; 3 Department of Orthopaedic Surgery, Tongji Hospital, Tongji University, Shanghai 200092, China

**Keywords:** early mobilization, internal fixation, proximal humeral fracture, rehabilitation, retrospective cohort study, shoulder function, valgus impacted fracture

## Abstract

**Objectives:** The optimal rehabilitation protocol following open reduction and internal fixation (ORIF) for Neer type-4 valgus impacted proximal humeral fractures (VIPHF) remains controversial. This study aimed to compare the long-term efficacy and safety of early active mobilization versus conventional rehabilitation in these patients. **Methods:** We conducted a retrospective cohort study at a single tertiary hospital involving 128 patients who underwent ORIF for Neer type-4 VIPHF between January 2018 and December 2022. Participants were divided into two groups based on the rehabilitation protocol received: the Early Active Activation (EAA) group (*n* = 64), which initiated controlled active exercises on postoperative day 1, and the Conventional Rehabilitation (CR) group (*n* = 64), which followed a delayed protocol. The primary outcome was the Constant–Murley score at 24 months. Secondary outcomes included scores at 3, 6, and 12 months, pain VAS scores, active range of motion, fracture healing time, and complications. **Results:** The EAA group demonstrated significantly superior outcomes, including a higher 24-month Constant–Murley score (88.7 ± 6.5 vs. 75.3 ± 9.2, *p* < 0.001), lower VAS scores at all time points (*p* < 0.001), greater range of motion (*p* < 0.001), and shorter fracture healing time (10.2 ± 1.8 vs. 12.5 ± 2.3 weeks, *p* < 0.001). Complication rates did not differ significantly (4.7% vs. 6.3%, *p* = 0.718). **Conclusions:** For patients with surgically stabilized Neer type-4 VIPHF, early active rehabilitation initiated on postoperative day 1 was associated with significantly improved functional outcomes, accelerated recovery, and a favorable safety profile. However, these findings should be interpreted with caution due to the retrospective design, single-center setting, and potential residual confounding. Further validation through prospective, multicenter studies is recommended.

## 1. Introduction

Proximal humeral fractures represent a significant clinical burden, particularly in osteoporotic elderly populations, with epidemiological studies indicating they constitute approximately 4–5% of all skeletal fractures [1]. Among these, complex fracture patterns such as four-part fractures present a particular therapeutic challenge. The management of severe fractures, whether in the hip or the proximal humerus, often hinges on achieving stable fixation to facilitate early rehabilitation or on opting for arthroplasty when the joint is deemed unsalvageable [2]. Specifically, the Neer type-4 valgus impacted proximal humeral fracture (VIPHF) represents a unique subset of these complex injuries. Unlike standard four-part fractures, VIPHF is characterized by preservation of the medial hinge, reduced risk of avascular necrosis, making it a distinct entity for which internal fixation remains a viable and often preferred options [3,4]. However, even with stable fixation, the optimal postoperative rehabilitation pathway is not well-defined.

Despite improvements in implant design and surgical techniques, functional recovery after ORIF for these fractures remains challenging. Common complications such as shoulder stiffness, muscle atrophy, and persistent pain significantly compromise patients’ quality of life [5,6]. Traditional postoperative management has often favored a cautious approach, prioritizing immobilization in the initial weeks to protect the fixation [7]. Active strengthening and mobility exercises are only initiated after radiographic evidence of early healing is observed. Emerging evidence, however, suggests that prolonged unloading may inadvertently predispose patients to adverse outcomes such as periarticular fibrosis and muscular atrophy [8].

The concept of Enhanced Recovery After Surgery (ERAS) has gained increasing attention in orthopedics, promoting evidence-based perioperative interventions to minimize surgical stress and accelerate recovery [9]. Within this framework, the feasibility and safety of early active mobilization following ORIF for Neer type-4 VIPHF require further evaluation. Although early motion may theoretically prevent stiffness, enhance cartilage nutrition, and preserve muscle strength, concerns remain regarding potential risks such as hardware failure, screw perforation, and fracture displacement.

Currently, there is a lack of high-quality evidence regarding the long-term benefits and risks of structured early active rehabilitation initiated on postoperative day 1 in patients with Neer type-4 VIPHF. Therefore, the primary objective of this retrospective cohort study was to compare the long-term functional outcomes and safety of an early active mobilization protocol versus a conventional rehabilitation protocol in patients with operatively treated Neer type-4 VIPHF. We hypothesized that early active mobilization initiated on postoperative day 1 would lead to significantly better long-term shoulder function without increasing the risk of complications.

## 2. Materials and Methods

### 2.1. Study Design

A consecutive sampling method was employed. All patients who underwent ORIF for proximal humeral fractures (VIPHF) at our institution between January 2018 and December 2022 were screened for eligibility. Those who met the inclusion criteria were enrolled in chronological order of their surgical dates until the sample size was reached. The study was approved by the hospital ethics committee (Approval No: T24-172). The need for informed consent was waived by the Institutional Review Board due to the retrospective nature of the study and the use of fully anonymized clinical data. This study was conducted in accordance with the Declaration of Helsinki.

### 2.2. Patient Selection

Inclusion criteria were age ≥18 years; diagnosis of Neer type-4 valgus impacted fracture confirmed by preoperative X-ray and 3D CT reconstruction; ORIF performed using the same proximal humeral locking plate system (Double Medical, Xiamen, China) by the same team of senior surgeons; and complete clinical and imaging data with a postoperative follow-up of at least 24 months.

Exclusion criteria included pathological fracture or concomitant ipsilateral upper limb fracture; delayed presentation (>3 weeks from injury to surgery); combined brachial plexus injury; previous ipsilateral shoulder surgery or pre-existing shoulder dysfunction; and severe cardiac, pulmonary, neurological, or other medical conditions that would interfere with compliance to rehabilitation.

### 2.3. Grouping Method

Based on the actual postoperative rehabilitation protocol received, patients were divided into two groups: the Early Active Activation (EAA) group and the Conventional Rehabilitation (CR) group. All patients received the same multimodal analgesia protocol to ensure feasibility of rehabilitation exercises.

### 2.4. Rehabilitation Protocol

#### 2.4.1. Early Active Activation (EAA) Group

The rehabilitation protocol for the EAA group was based on the concept of initiating controlled active movement immediately after surgery, aiming to promote venolymphatic return, reduce swelling, and prevent joint stiffness and muscle atrophy through active muscle contraction. The protocol was implemented in three phases:

Phase 1 (Postoperative Days 1–2 weeks): Maximum Protection Phase

The focus was on pain and swelling reduction, maintaining range of motion (ROM) of adjacent joints, and initiating early active shoulder movement.

Activities included:

- Active shoulder flexion and abduction within a pain-free range, performed slowly and smoothly without momentum; multiple daily sessions of 10–15 repetitions each.
- Use of a shoulder abduction pillow or sling for comfort during rest.
- Active full-range wrist and elbow flexion/extension exercises and hand grip training.
- Pendulum exercises 3–4 times daily for 5–10 min per session.
- Assisted flexion/abduction exercises using the unaffected arm, with goals of achieving 90° within one week and 120° within two weeks.
- Pulley-assisted training when feasible.

Phase 2 (Weeks 3–6): Moderate Protection Phase

Emphasis was placed on increasing active ROM and enhancing proprioception and coordination. Patients continued and intensified Phase 1 exercises while gradually reducing assistance and increasing active movement. Goals included achieving ≥150° active flexion and ≥120° active abduction by Week 6.

Additional training included:

- Gentle internal and external rotation exercises in supine position with the elbow flexed at 90°.
- Scapular stabilization exercises (retraction, protraction, elevation, and depression) without resistance.

Phase 3 (Weeks 7–24): Functional Recovery Phase

The focus shifted to restoring full joint ROM, initiating strength training, and ultimately resuming daily activities. After achieving full pain-free ROM and confirming radiographic healing, patients began strength training starting with isometric contractions and progressing to low-load resistance exercises. Functional training (e.g., wall climbing, hair combing) and endurance/coordination exercises were incorporated.

Throughout all phases, the protocol emphasized individualized adjustment and avoidance of high-risk movements, ensuring exercises were performed within a tolerable pain range.

#### 2.4.2. Conventional Rehabilitation (CR) Group

The rehabilitation protocol for the CR group adhered to the core principle of strictly protecting the internal fixation in the early postoperative period, emphasizing maintenance of joint range of motion through passive activities. Active motion and strength training were gradually introduced only after preliminary fracture healing was confirmed. The protocol was carried out in three phases:

Phase 1 (Postoperative Weeks 0–6): Strict Immobilization Phase

The focus was on protecting the fixed structures, reducing pain and swelling, and maintaining passive joint mobility.

Specific measures included

- Continuous use of a shoulder abduction pillow or sling.
- Non-immobilized joint exercises and pendulum exercises identical to those in the EAA group.
- Strict passive range of motion (PROM) training, where the therapist or the patient’s unaffected hand passively performed shoulder flexion, abduction, and external rotation within a pain-free range not exceeding 90° for flexion/abduction and neutral position for external rotation.
- Any form of active muscle contraction or anti-gravity movement was strictly prohibited.

Phase 2 (Weeks 7–12): Active Motion Transition Phase

After radiographic confirmation of continuous callus formation, patients transitioned from passive to active-assisted movement.

Measures included

- Gradually reducing sling use.
- Initiating active-assisted range of motion (AAROM) exercises identical to those in Phase 1 of the EAA group.
- Progressive introduction of active range of motion (AROM) training starting in anti-gravity positions (e.g., supine), advancing to seated and standing positions as tolerated.

Phase 3 (Week 13 and Beyond): Functional and Strength Recovery Phase

The primary goals were restoring full range of motion and improving muscle strength and endurance. Patients performed active and resistance training largely similar to Phase 3 of the EAA group. However, due to the delayed initiation of active movement, this phase typically required longer and more intensive effort to address joint stiffness and muscle atrophy.

Throughout rehabilitation, the process emphasized gradual progression, with advancement strictly guided by radiographic evidence of fracture healing.

### 2.5. Outcome Measures

**Primary outcome measure:** Shoulder function assessed using the Constant–Murley score (total score 100, encompassing pain, activities of daily living, range of motion, and muscle strength) at 24 months postoperatively.

**Secondary outcome measures included**

- Functional scores: Constant–Murley scores at 3, 6, and 12 months postoperatively.
- Pain assessment: Visual Analog Scale (VAS) scores at 3, 6, 12, and 24 months postoperatively.
- Range of motion (ROM): Active flexion, abduction, and external rotation of the affected shoulder at each postoperative time point, measured using a goniometer.
- Fracture healing time: Defined as the presence of continuous callus formation across the fracture site on radiographs and the absence of pain upon weight-bearing.
- Complications: Incidence of events such as implant failure (e.g., plate breakage, screw loosening), screw cut-out, fracture redisplacement, avascular necrosis (AVN) of the humeral head, and infection.
- Assessment of AVN Assessment: Standard Radiographs: Obtained at each follow-up visit (6 weeks, 3, 6, 12, and 24 months postoperatively) for initial screening. CT Confirmation: Performed with 3D reconstruction when radiographs suggested AVN (increased density, subchondral collapse, or segmental depression of the humeral head). Detailed information on observer training and the results of intra- and inter-observer reliability analyses are provided in the Supplementary Materials (Observer Training and Reliability Assessment Protocol; Table S1).

### 2.6. Statistical Analysis

Data analysis was performed using SPSS software (version 23.0). Continuous variables (e.g., Constant score, VAS score, ROM) were expressed as mean ± standard deviation ($\bar{x}$ ± s) and compared between groups using independent samples *t*-tests. For longitudinal data (Constant–Murley scores, VAS scores, and ROM across multiple time points), a two-way repeated-measures analysis of variance (RM-ANOVA) was employed with Time as the within-subjects factor and Group as the between-subjects factor. The primary effect of interest was the Group × Time interaction. If a significant interaction was found, it would indicate that the recovery trajectory differed between groups. Post hoc pairwise comparisons, adjusted for multiple comparisons using the Bonferroni method, were then used to identify specific between-group differences at each time point. Categorical variables were summarized as counts (percentages) and compared using chi-square tests or Fisher’s exact test. A two-sided *p*-value < 0.05 was considered statistically significant.

### 2.7. Control of Bias

Due to the retrospective design, potential biases were addressed through several measures. Predefined inclusion/exclusion criteria enhanced cohort homogeneity. Although baseline characteristics were balanced, unmeasured confounders (e.g., patient motivation and compliance) may persist. Multivariate regression adjusted for available covariates (age, gender, comorbidities), and sensitivity analysis explored unmeasured confounding. Standardized instruments (Constant–Murley, VAS) minimized measurement bias. Despite these efforts, residual confounding inherent to observational studies should be considered when interpreting the results. To assess the robustness of our primary findings, we conducted extensive sensitivity analyses. The results of these analyses, which evaluate the potential impact of unmeasured confounding, are presented in Table S2 of the Supplementary Materials. Additionally, subgroup analyses of the primary outcome are shown in Table S3.

## 3. Results

**Figure 1:**
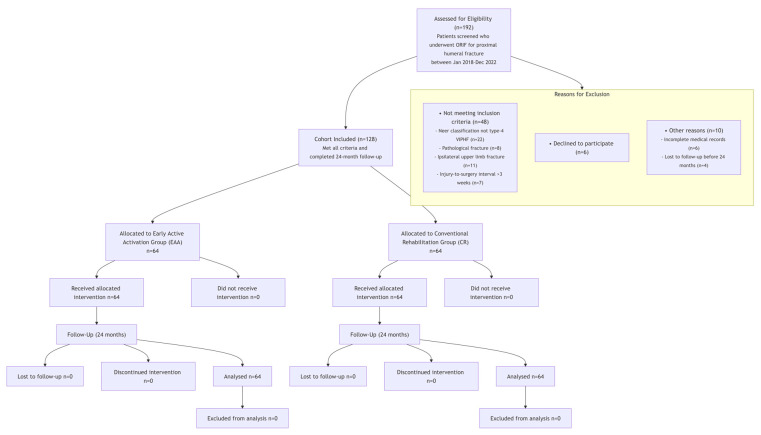
Patient Flow Diagram.

Due to the retrospective nature of this study, a formal prospective sample size calculation was not performed. Instead, we employed a comprehensive sampling approach by including all consecutive patients who met the eligibility criteria during the five-year study period (January 2018 to December 2022). During the study period, 192 patients underwent ORIF for proximal humeral fractures. After assessment against the inclusion and exclusion criteria, 128 patients were enrolled in the final analysis, as detailed in (Figure 1). A total of 128 patients who completed the 24-month follow-up were ultimately included in this study, with 64 patients each in the Early Active Activation (EAA) group and the Conventional Rehabilitation (CR) group. No missing data occurred for any primary or secondary outcomes, and thus no data imputation was required. The total sample size provides adequate statistical power for our primary analysis. Based on a post hoc power analysis with an effect size of 1.2 (derived from the observed difference in Constant–Murley scores), alpha of 0.05, and sample size of 64 per group, our study achieved a statistical power of 99%, substantially exceeding the conventional threshold of 80%.

The baseline characteristics of the two groups are presented in Table 1. There were no statistically significant differences in age, gender, fracture side, time from injury to surgery, or comorbidities between the two groups (*p* > 0.05), indicating that the groups were comparable. Subgroup analyses based on age and gender showed results consistent with the primary analysis (see Table S3).

Functional assessment results showed that the Constant–Murley scores of the EAA group were significantly better than those of the CR group at all postoperative time points (Table 2). For the primary outcome measure, the score at 24 months postoperatively was significantly higher in the EAA group (88.7 ± 6.5) than in the CR group (75.3 ± 9.2) (mean difference: 13.4 points; 95% CI: 10.6 to 16.2; *p* < 0.001). Functional recovery over time showed that as early as 3 months postoperatively, the EAA group already achieved a score of 62.5 ± 7.3, which was significantly higher than the CR group’s 45.8 ± 8.6 (*p* < 0.001). This advantage persisted at 6 months (76.3 ± 6.8 vs. 58.9 ± 9.1) and 12 months (82.4 ± 6.2 vs. 68.7 ± 8.9).

Pain assessment results indicated that the VAS scores in the EAA group were significantly lower than those in the CR group at all postoperative time points (Table 3). At 3 months postoperatively, the VAS score in the EAA group (2.8 ± 0.9) was already significantly lower than that in the CR group (4.2 ± 1.3) (*p* < 0.001). This advantage in pain relief continued at 6 months (2.1 ± 0.7 vs. 3.5 ± 1.1), 12 months (1.5 ± 0.6 vs. 2.8 ± 1.0), and 24 months (1.2 ± 0.5 vs. 2.3 ± 0.9).

Regarding joint range of motion (Table 4), at 3 months postoperatively, the active flexion, abduction, and external rotation angles in the EAA group were 92.5 ± 10.3°, 88.7 ± 11.2°, and 28.6 ± 6.8°, respectively, which were significantly greater than those in the CR group (68.7 ± 12.6°, 62.4 ± 13.8°, and 18.3 ± 7.5°). Over time, the range of motion improved progressively in both groups, but the extent of recovery was consistently greater in the EAA group at all time points. At the 24-month follow-up, the EAA group showed significantly greater active range of motion in all planes measured: flexion (mean difference: 25.7°, 95% CI: 20.4 to 31.0), abduction (mean difference: 27.3°, 95% CI: 21.4 to 33.2), and external rotation (mean difference: 7.1°, 95% CI: 3.8 to 10.4; all *p* < 0.001; Table 4).

Furthermore, regarding the fracture healing process, the EAA group demonstrated significantly faster healing. The radiographically assessed clinical fracture healing time was 10.2 ± 1.8 weeks in the EAA group, significantly shorter than the 12.5 ± 2.3 weeks in the CR group (t = 6.32, *p* < 0.001). This suggests that early active mobilization not only improved joint function but may also have enhanced the biological process of fracture healing.

In terms of safety, there was no statistically significant difference in the overall complication rate between the two groups (4.7% in the EAA group vs. 6.3% in the CR group, *p* = 0.718). The EAA group had one case of screw cut-out and two cases of mild fracture displacement; the CR group had one case of internal fixation failure, two cases of screw cut-out, and one case of superficial infection. All complications were managed appropriately with corresponding treatments, and no symptomatic avascular necrosis of the humeral head occurred in either group (Table 5).

To address potential confounding and selection bias, a multivariate linear regression analysis was performed, adjusting for age, gender, and comorbidities. The results confirmed that the rehabilitation group (EAA vs. CR) was an independent predictor of the 24-month Constant–Murley score (β = 12.8, 95% CI: 9.9 to 15.7, *p* < 0.001), even after accounting for these covariates.

Additionally, a sensitivity analysis was conducted to quantify the potential impact of an unmeasured confounder (e.g., patient motivation) on the primary outcome. The results suggest that an unmeasured confounder would need to have a strong association with both the group assignment and the functional outcome (e.g., risk ratio > 2.5) to fully explain the observed treatment effect, which is considered unlikely in clinical practice. Furthermore, a 1:1 propensity score matching (PSM) was performed to enhance the comparability of the groups. The propensity score was generated using a logistic regression model that included all available baseline covariates. After matching (caliper = 0.05), 58 well-balanced pairs were created, with all standardized mean differences (SMDs) for covariates reduced to <0.1, indicating successful balancing. In this matched cohort, the results remained robust, with the EAA group demonstrating a significantly higher Constant–Murley score than the CR group (87.9 ± 6.1 vs. 74.8 ± 8.9, *p* < 0.001).

**Figure 2:**
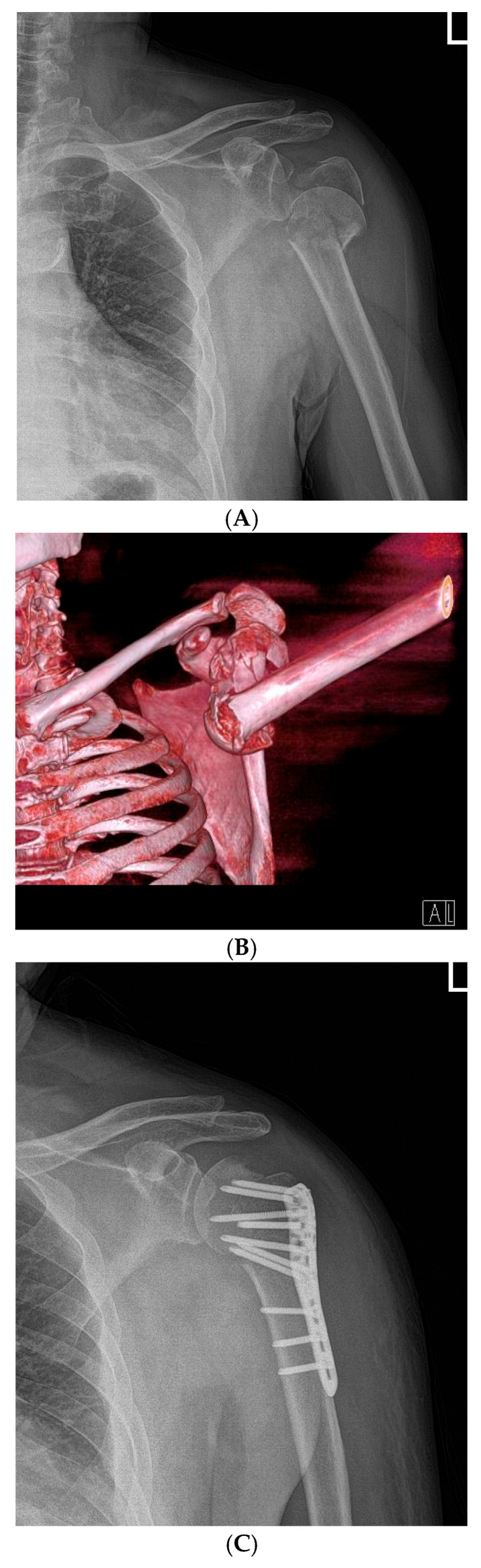

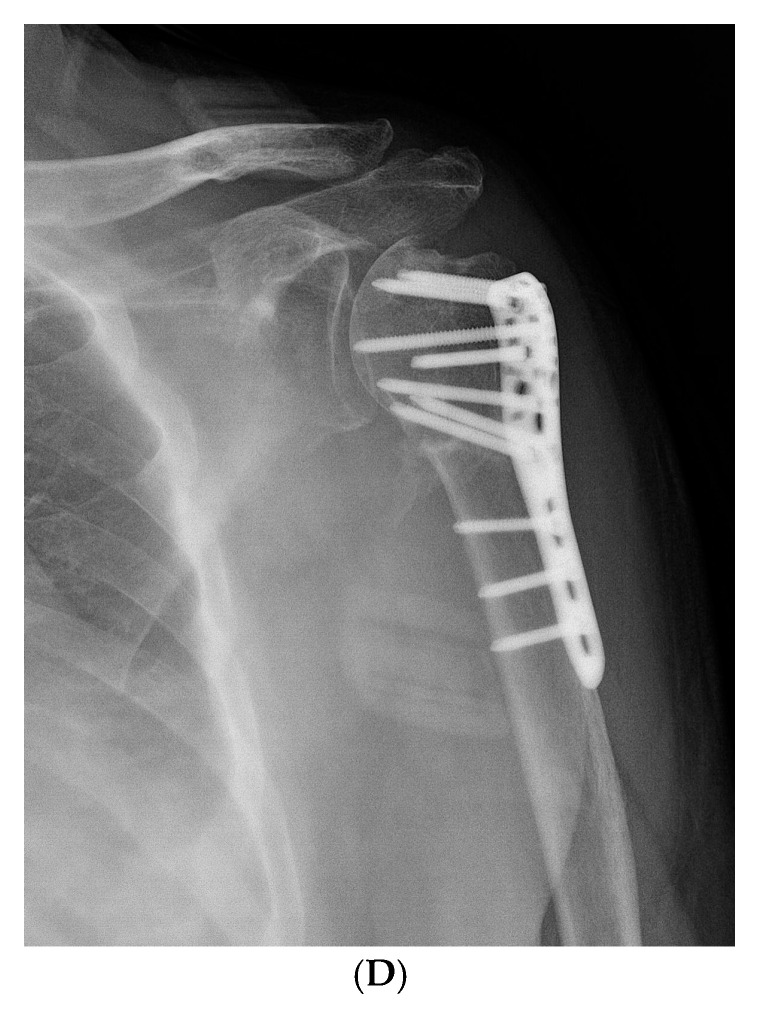
Representative Case 1: 68-year-old female with Neer type-4 valgus impacted proximal humeral fracture treated with open reduction and internal fixation (ORIF) and early active rehabilitation. (**A**) Preoperative anteroposterior (AP) radiograph showing valgus impacted four-part fracture with medial hinge integrity preserved. (**B**) Preoperative 3D CT reconstruction confirming fracture pattern and displacement. (**C**) Immediate postoperative AP radiograph demonstrating anatomical reduction and stable fixation with proximal humeral locking plate (Double Medical, Xiamen, China). (**D**) 24-month follow-up AP radiograph showing complete fracture union with well-maintained reduction, no evidence of avascular necrosis or hardware complications.

**Figure 3:**
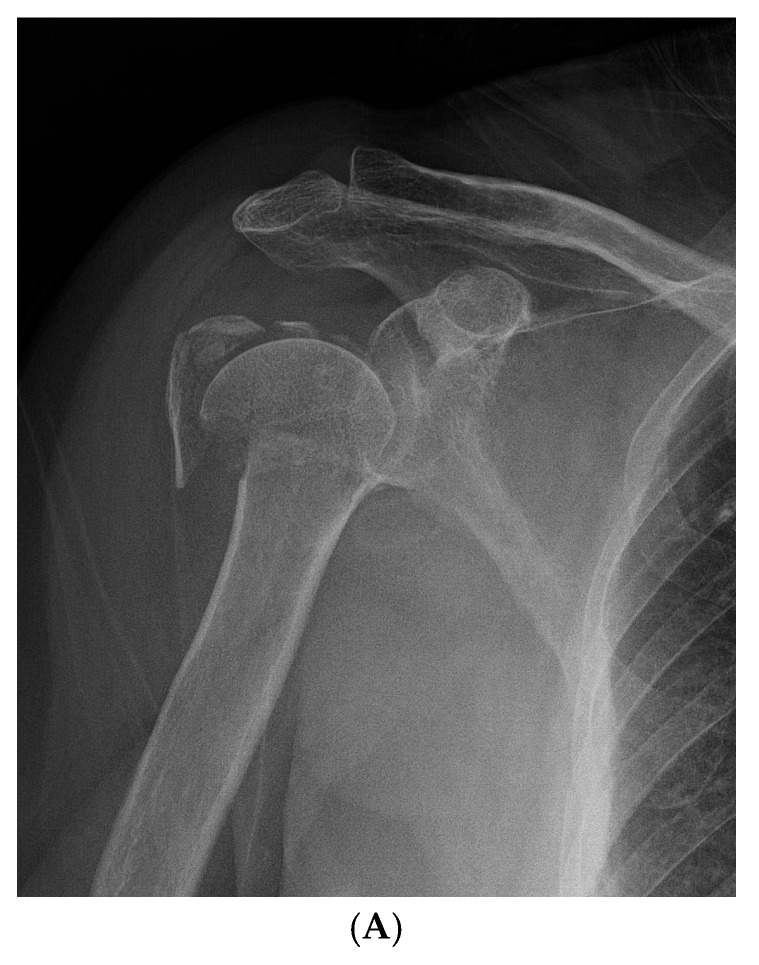

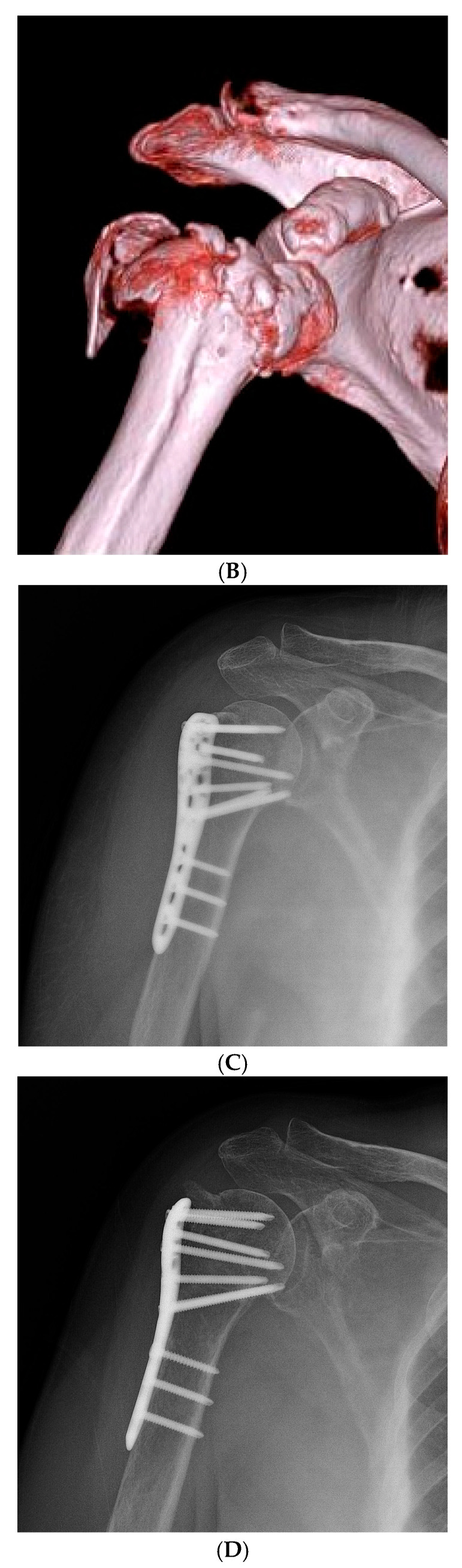
Representative Case 2: 58-year-old male with Neer type-4 valgus impacted proximal humeral fracture treated with ORIF and conventional rehabilitation. (**A**) Preoperative AP radiograph showing severely displaced valgus impacted fracture. (**B**) Preoperative axial CT scan illustrating fragment displacement and rotation. (**C**) Immediate postoperative AP radiograph showing satisfactory reduction and internal fixation. (**D**) 24-month follow-up AP radiograph demonstrating complete fracture healing, and maintained functional range of motion.

In summary, early postoperative active mobilization significantly improves shoulder function scores, reduces pain, increases range of motion, and promotes fracture healing in patients with Neer type-4 VIPHF (Figure 2 and Figure 3), without increasing the risk of complications.

### Missing Data Handling

Due to the rigorous follow-up protocol implemented in this study, complete data were obtained for all 128 enrolled participants at all assessment time points (3, 6, 12, and 24 months). No missing data occurred in the primary outcome (24-month Constant–Murley score) or any secondary outcomes. As such, no imputation methods were required, and all analyses were performed using complete-case analysis.

## 4. Discussion

This retrospective cohort study systematically evaluated the impact of early postoperative active mobilization on long-term functional recovery in patients with Neer type-4 valgus impacted proximal humeral fractures (VIPHF) treated with internal fixation. The results demonstrated that the experimental group, which began instructed active range-of-motion training on the first postoperative day, achieved significantly better outcomes at 24 months compared to the control group following a traditional delayed active rehabilitation protocol. These improvements were reflected in the Constant–Murley score, VAS pain score, and active range of motion (flexion, abduction, and external rotation). Furthermore, early mobilization did not increase the risk of complications such as implant failure or screw cut-out and even showed a trend toward promoting fracture healing (healing time: 10.2 ± 1.8 weeks vs. 12.5 ± 2.3 weeks). These findings indicate that early active mobilization is a safe and highly effective rehabilitation strategy for appropriately selected VIPHF patients.

### 4.1. Significance of Early Rehabilitation Exercise

In the rehabilitation of fracture patients, the timing of intervention is critical for functional prognosis and recovery efficacy. Existing studies generally define rehabilitation activities initiated within 24 h to one week after surgery as early rehabilitation [10,11]. During this period, the patient’s physiological condition is relatively stable, and the initial phase of tissue healing presents an optimal window for initiating functional exercise [12]. Studies have shown that early mobilization within 24 h post-surgery can significantly reduce the incidence of shoulder complications and help improve patients’ quality of life [10,12].

The timing and modality of rehabilitation introduce critical biological and mechanical stimuli that can directly influence the fracture healing cascade [13,14]. In the present study, early controlled motion was hypothesized to generate beneficial micromotion at the osteosynthesis site, potentially creating a more conducive microenvironment for bone repair. This is mechanistically supported by the application of controlled axial loading, which may enhance callus formation and remodeling through mechanotransduction pathways [15,16]. Our findings, showing significantly reduced healing time in the EAA group, provide clinical corroboration for this proposed mechanism.

The functional integrity of the shoulder girdle is inherently dependent on the synchronized kinematics of the glenohumeral, scapulothoracic, and clavicular articulations. Our rehabilitation protocol specifically incorporated exercises targeting scapular stabilization and rhythmic control based on the principle that re-establishing this kinetic chain synchrony is paramount for functional recovery. A key objective of initiating early, guided motion was to mitigate the risk of capsular hypomobility and facilitate the restoration of physiological arthrokinematics [17,18]. The biomechanical properties of the shoulder, including joint stability and range of motion, are influenced by early rehabilitation activities. Appropriate exercise not only promotes synovial fluid flow and reduces adhesion formation but also enhances shoulder function by strengthening the surrounding muscles [10]. Therefore, early rehabilitation should emphasize coordinated movement between the glenohumeral and scapulothoracic joints to achieve optimal functional recovery.

For patients with proximal humeral fractures, it is recommended to initiate early rehabilitation as soon as possible after surgery to facilitate functional recovery and reduce the incidence of complications during the initial stages of fracture healing.

### 4.2. Value of Early Active Shoulder Functional Exercise

The core significance of our findings lies in challenging the traditional notion that strict immobilization is necessary after surgical treatment of complex proximal humeral fractures. VIPHF possesses a unique biological advantage due to the relatively preserved blood supply to the humeral head (benefiting from the intact medial hinge) [2], which provides an essential anatomical basis for early functional activity. Active muscle contraction in the early stage not only effectively promotes local blood circulation and reduces pain and swelling but, more importantly, prevents joint capsule adhesion and disuse atrophy of the periarticular muscles—both of which are key causes of shoulder stiffness. In this study, the experimental group demonstrated significant functional advantages as early as 3 months postoperatively, which were sustained for up to 2 years, strongly supporting the critical importance of early intervention in avoiding stiffness and achieving long-term functional benefits.

It is important to note that the successful implementation of “early active mobilization” in this study relied on several key prerequisites: First, all surgeries were performed by an experienced surgical team using standardized internal fixation techniques, ensuring absolute stability of the initial fixation, which forms the mechanical foundation for early functional exercise. Second, rehabilitation training was conducted under the one-on-one guidance of a physiotherapist within a “controlled range,” strictly prohibiting resistance and strenuous activities to ensure safety. Furthermore, the effective implementation of a multimodal analgesia protocol created conditions for patients to overcome pain and adhere to rehabilitation. Therefore, the success of this protocol should not be simply interpreted as “the earlier, the better,” but rather emphasized as a systematic engineering effort supported by three pillars: “optimized fixation, professional guidance, and effective analgesia.”

Regarding safety, the lack of a statistically significant difference in the overall complication rate between the two groups demonstrates that early mobilization does not mechanically increase the risk of internal fixation failure when the above conditions are met. This aligns with the perspectives of some recent prospective studies [4], indicating that an “individualized accelerated rehabilitation” protocol based on fracture type and fixation stability is safe and feasible. Nevertheless, this study has limitations. As a retrospective study, despite efforts to control for baseline comparability, unmeasured confounding factors may still exist. Additionally, since all patients were from a single center, the generalizability of the conclusions requires further validation through multicenter, large-sample, prospective randomized controlled trials.

### 4.3. Limitations

Although our study demonstrated baseline comparability between groups, we strengthened the analytical rigor by performing multivariate regression adjusting for key potential confounders including age, sex, and comorbidity status. The consistency between adjusted and unadjusted results enhances our confidence that the observed functional improvements are indeed attributable to the rehabilitation protocol rather than these demographic factors.

Despite efforts to enhance the robustness of our findings through multivariate regression and sensitivity analyses, this study retains limitations inherent to its retrospective design. Although we controlled for available confounding variables, unmeasured factors, such as individual patient motivation, socioeconomic status, and precise compliance with rehabilitation protocols, may still influence functional outcomes. Furthermore, all participants were sourced from a single institution, which may affect the generalizability of our results. Although propensity score matching was performed to improve comparability, residual confounding cannot be entirely excluded. Future prospective, multicenter, randomized controlled trials are warranted to validate these findings and further establish causal relationships.

## 5. Conclusions

In conclusion, this single-center retrospective study demonstrates that for appropriately selected patients with Neer type-4 valgus impacted proximal humeral fractures (VIPHF) who have achieved stable internal fixation, implementing a controlled early active rehabilitation protocol starting on postoperative day 1 is associated with significantly improved long-term shoulder function, reduced pain levels, accelerated recovery timeline, and a favorable safety profile. These compelling findings challenge conventional immobilization-based rehabilitation approaches and indicate that early mobilization represents a promising therapeutic strategy in suitable candidates. However, given the inherent limitations of the retrospective design and single-center setting, the application of this protocol should be limited to settings with strict patient selection criteria, standardized surgical techniques, and supervised rehabilitation environments. We strongly recommend that these findings be validated through large-scale, multicenter, prospective randomized controlled trials before any widespread clinical implementation can be considered.

## Figures and Tables

**Table 1:** Comparison of baseline characteristics between groups.

Indicator	EAA Group	CR Group	*p*-Value
Age (years)	62.3 ± 8.7	63.1 ± 9.2	0.621
Gender (Male/Female)	25/39	28/36	0.592
Affected Side (Left/Right)	29/35	32/32	0.655
Time from Injury to Surgery (days)	4.2 ± 1.5	4.5 ± 1.8	0.307
Hypertension (*n*, %)	22 (34.4%)	25 (39.1%)	0.703

**Table 2:** Comparison of Constant–Murley Scores at Different Postoperative Time Points (score, $\bar{x}$ ± s).

Time Point	EAA Group	CR Group	Mean Difference (95% CI)	*p*-Value
3 months	62.5 ± 7.3	45.8 ± 8.6	16.7 (13.8 to 19.6)	<0.001
6 months	76.3 ± 6.8	58.9 ± 9.1	17.4 (14.6 to 20.2)	<0.001
12 months	82.4 ± 6.2	68.7 ± 8.9	13.7 (11.0 to 16.4)	<0.001
24 months	88.7 ± 6.5	75.3 ± 9.2	13.4 (10.6 to 16.2)	<0.001

**Table 3:** Comparison of VAS Scores at Different Postoperative Time Points (score, $\bar{x}$ ± s).

Time Point	EAA Group	CR Group	Mean Difference (95% CI)	*p*-Value
3 months	2.8 ± 0.9	4.2 ± 1.3	−1.4 (−1.8 to −1.0)	<0.001
6 months	2.1 ± 0.7	3.5 ± 1.1	−1.4 (−1.7 to −1.1)	<0.001
12 months	1.5 ± 0.6	2.8 ± 1.0	−1.3 (−1.6 to −1.0)	<0.001
24 months	1.2 ± 0.5	2.3 ± 0.9	−1.1 (−1.4 to −0.8)	<0.001

**Table 4:** Comparison of Active Shoulder Range of Motion at Different Postoperative Time Points (degrees, $\bar{x}$ ± s).

Motion	Group	3 Months	6 Months	12 Months	24 Months
Flexion	EAA Group	92.5 ± 10.3	125.8 ± 12.7	142.3 ± 11.5	158.5 ± 12.3
	CR Group	68.7 ± 12.6	96.4 ± 14.2	118.9 ± 13.8	132.8 ± 16.7
	*p*-value	<0.001	<0.001	<0.001	<0.001
Abduction	EAA Group	88.7 ± 11.2	118.6 ± 13.5	136.8 ± 12.4	152.6 ± 14.2
	CR Group	62.4 ± 13.8	90.2 ± 15.7	112.5 ± 15.3	125.3 ± 18.4
	*p*-value	<0.001	<0.001	<0.001	<0.001
External Rotation	EAA Group	28.6 ± 6.8	36.5 ± 7.9	41.8 ± 8.3	45.3 ± 8.6
	CR Group	18.3 ± 7.5	26.7 ± 8.4	33.4 ± 9.1	38.2 ± 10.5
	*p*-value	<0.001	<0.001	<0.001	<0.001

**Table 5:** Comparison of Complication Rates.

Complication Type	EAA Group	CR Group	*p*-Value
Implant Failure	0	1 (1.6%)	1
Screw Cut-out	1 (1.6%)	2 (3.1%)	1
Fracture Redisplacement	2 (3.1%)	0	0.497
Avascular Necrosis	0	0	-
Infection	0	1 (1.6%)	1
**Overall Complications**	3 (4.7%)	4 (6.3%)	0.718

## Data Availability

The anonymized dataset generated and analyzed during this study is not publicly available to protect patient privacy. However, it will be made available from the corresponding author upon reasonable request. Requests should include a detailed research proposal outlining the intended use of the data and will be subject to approval by the institutional ethics committee. Data will be shared under a data use agreement that includes commitments to using the data only for the specified research purpose, protecting data confidentiality, and not attempting to identify individual participants.

## Supplementary Materials

The following supporting information can be downloaded at: https://www.mdpi.com/article/10.3390/jcm14186660/s1, Method S1: Observer Training and Reliability Assessment Protocol; Table S1. Intra-observer and Inter-observer Reliability for Radiographic Measurements; Table S2. Results of Sensitivity Analyses Evaluating the Robustness of the Primary Outcome; Table S3. Subgroup Analyses of the Primary Outcome (24-Month Constant–Murley Score).
