# Supplementary material for: The Value of Early Active Rehabilitation on Long-Term Functional Outcomes in Neer Type-4 Valgus Impacted Proximal Humeral Fractures Treated with Open Reduction and Internal Fixation: A Retrospective Cohort Study
Source: J Clin Med. 2025 Sep 22;14(18):6660. doi: 10.3390/jcm14186660 (PMC12470890; doi:10.3390/jcm14186660)
Supplement: Supplementary file 1 [file jcm-14-06660-s001.zip › jcm-3862887-supplementary.pdf]

Supplementary Method S1.
Observer Training and Reliability Assessment Protocol

Training Protocol:

Prior to formal radiographic measurements, two board-certified orthopedic surgeons with >5 years of musculoskeletal imaging experience underwent a standardized training session. The training included

1. **Theoretical Review:** A 2-hour session covering the definitions of all radiographic parameters (e.g., head-shaft angle, greater tuberosity displacement, fracture healing criteria, signs of AVN) based on established orthopedic references.
2. **Practical Calibration:** Independent assessment of 20 sample cases (not included in the study cohort) with various fracture patterns and healing stages. Each observer measured all parameters twice, with a 2-week interval between assessments.
3. **Consensus Meeting:** A joint review of all sample cases with a senior musculoskeletal radiologist (15 years of experience) to resolve discrepancies and establish uniform measurement standards.

Reliability Assessment:

Following training, both observers independently measured all radiographic parameters for the first 30 patients in the study cohort. Measurements were repeated after a 2-week washout period, with images presented in random order.

Statistical Analysis:

Intra-observer and inter-observer reliability were assessed using two-way random-effects intraclass correlation coefficients (ICC) for continuous variables (e.g., displacement measurements) and Cohen's kappa (κ) for categorical variables (e.g., fracture union, AVN diagnosis). ICC and κ values were interpreted as follows: <0.40 poor; 0.40-0.59 fair; 0.60-0.74 good; ≥0.75 excellent.

Supplementary Table S1. Intra-observer and Inter-observer Reliability for Radiographic Measurements

Parameter	Intra-observer Reliability (ICC/κ)	Inter-observer Reliability (ICC/κ)
**Head-shaft angle (°)**	0.92 (0.87-0.95)	0.89 (0.82-0.93)
**Greater tuberosity displacement (mm)**	0.88 (0.79-0.93)	0.85 (0.75-0.91)
**Humeral head height (mm)**	0.91 (0.85-0.95)	0.87 (0.78-0.92)
**Fracture union (κ)**	0.82 (0.71-0.90)	0.78 (0.66-0.87)
**AVN diagnosis (κ)**	0.79 (0.67-0.88)	0.75 (0.62-0.85)

Data presented as ICC/κ value (95% confidence interval). All values showed excellent reliability (≥0.75).*

Abbreviations: ICC, intraclass correlation coefficient; κ, kappa coefficient; AVN, avascular necrosis.

Supplementary Table S2. Results of Sensitivity Analyses Evaluating the Robustness of the Primary Outcome

Analysis Method	Comparison	Effect Size (95% CI)	P-value	Conclusion
**Primary Analysis (t-test)**	EAA vs. CR Group	13.4 (10.6 to 16.2)	<0.001	Significant difference favoring EAA group
**Multivariate Regression**	Adjusted for age, sex, hypertension	12.8 (9.9 to 15.7)	<0.001	Effect remains significant after adjustment for measured confounders
**Propensity Score Matched Cohort(n=58 pairs)**	EAA vs. CR Group	13.1 (10.1 to 16.1)	<0.001	Significant difference persists in a matched sample
**E-value Calculation**				
- For point estimate		2.8		An unmeasured confounder would need to be associated with both the group allocation and outcome by a risk ratio of 2.8-fold each to explain away the effect
- For CI lower limit		2.3		

Abbreviations: EAA, Early Active Activation group; CR, Conventional Rehabilitation group; CI, confidence interval.

The primary outcome was the Constant–Murley score at 24 months. The effect size represents the mean difference in scores between groups.

The propensity score was estimated using logistic regression with age, gender, and hypertension as covariates.

The E-value quantifies the minimum strength of association an unmeasured confounder would need to have to fully explain the observed effect.

Supplementary Table S3. Subgroup Analyses of the Primary Outcome (24-Month Constant–Murley Score)

Subgroup	No. of Patients (EAA/CR)	Constant–Murley Score, Mean ± SD	Mean Difference (EAA - CR)	95% CI	P-value
Overall	128 (64/64)	88.7 ± 6.5 vs. 75.3 ± 9.2	13.4	10.6 to 16.2	<0.001
Age < 65 years	68 (35/33)	89.5 ± 5.8 vs. 76.8 ± 8.5	12.7	9.2 to 16.2	<0.001
Age ≥ 65 years	60 (29/31)	87.7 ± 7.2 vs. 73.7 ± 9.8	14	10.1 to 17.9	<0.001
Male	53 (25/28)	89.2 ± 6.8 vs. 76.1 ± 8.7	13.1	9.2 to 17.0	<0.001
Female	75 (39/36)	88.3 ± 6.3 vs. 74.6 ± 9.6	13.7	10.4 to 17.0	<0.001
With Hypertension	47 (22/25)	87.9 ± 7.1 vs. 74.2 ± 10.3	13.7	9.3 to 18.1	<0.001
Without Hypertension	81 (42/39)	89.1 ± 6.1 vs. 76.0 ± 8.4	13.1	10.1 to 16.1	<0.001

Abbreviations: EAA, Early Active Activation group; CR, Conventional Rehabilitation group; CI, confidence interval; SD, standard deviation.

The mean difference, 95% CI, and p-value are derived from independent samples t-test for each subgroup.

All subgroup analyses demonstrated consistently superior outcomes for the EAA group, supporting the robustness of the primary findings.
